# Outpatient costs in pharmaceutically treated diabetes patients with and without a diagnosis of depression in a Dutch primary care setting

**DOI:** 10.1186/1472-6963-12-46

**Published:** 2012-02-23

**Authors:** Judith E Bosmans, Marcel C Adriaanse

**Affiliations:** 1 Section of Health Economics & Health Technology Assessment, Department of Health Sciences and EMGO Institute for Health and Care Research, VU University Amsterdam, Amsterdam, the Netherlands; 2 Section of Prevention and Public Health, Department of Health Sciences and EMGO Institute for Health and Care Research, VU University Amsterdam, Amsterdam

## Abstract

**Background:**

To assess differences in outpatient costs among pharmaceutically treated diabetes patients with and without a diagnosis of depression in a Dutch primary care setting.

**Methods:**

A retrospective case control study over 3 years (2002-2004). Data on 7128 depressed patients and 23772 non-depressed matched controls were available from the electronic medical record system of 20 general practices organized in one large primary care organization in the Netherlands. A total of 393 depressed patients with diabetes and 494 non-depressed patients with diabetes were identified in these records. The data that were extracted from the medical record system concerned only outpatient costs, which included GP care, referrals, and medication.

**Results:**

Mean total outpatient costs per year in depressed diabetes patients were €1039 (SD 743) in the period 2002-2004, which was more than two times as high as in non-depressed diabetes patients (€492, SD 434). After correction for age, sex, type of insurance, diabetes treatment, and comorbidity, the difference in total annual costs between depressed and non-depressed diabetes patients changed from €408 (uncorrected) to €463 (corrected) in multilevel analyses. Correction for comorbidity had the largest impact on the difference in costs between both groups.

**Conclusions:**

Outpatient costs in depressed patients with diabetes are substantially higher than in non-depressed patients with diabetes even after adjusting for confounders. Future research should investigate whether effective treatment of depression among diabetes patients can reduce health care costs in the long term.

## Background

Depression is common in primary care with prevalence rates ranging from 5% to 10% [1] and has a substantial impact on quality of life and societal costs [2,3]. In people with diabetes increased depression rates are found in comparison with people without diabetes [4].

Depression in combination with diabetes as compared with diabetes alone, has been linked with poor self-care and adherence to medical treatment,[5] poorer glycemic control,[6] more diabetes complications,[7] and a higher risk of morbidity and all cause mortality [8]. Moreover, comorbid depression and diabetes is associated with increased health care costs in comparison with diabetes alone [9,10].

Most depressed patients and diabetes patients are treated in primary care. In a recent retrospective study, we showed that diabetes was almost 3 times as prevalent in treated depressed patients than in matched controls in primary care [11]. Since both the numbers of patients with depression and of patients with diabetes are increasing rapidly worldwide and both conditions are associated with raised health care costs, it is important to examine the relation between depression, diabetes and costs.

Several studies determined the prevalence of depression in diabetes populations and subsequently evaluated costs [9,10]. In a large USA sample of adult health plan members with diabetes, depression was associated with 50-75% increase in health services use [9]. In a primary-care-based sample of patients with type 1 and 2 diabetes, higher levels of depressive symptom severity were associated with increased health care costs compared with patients with low-severity depressive symptoms [10]. Whereas the majority of other studies assessed the presence of depressive symptoms using questionnaires in a diabetes population, our study is unique in the sense that it was done in a very large primary care database using selection criteria based on objective information registered by the GP. The aim of the study was to compare differences in resource use and health care costs between pharmaceutically treated diabetes patients with and without depression who were identified in a primary care cohort from the Netherlands while correcting for age, sex and comorbidity.

## Methods

### Design

This was a retrospective case control study over 3 years (2002-2004). In a cohort of depressed patients and their non-depressed matched controls diabetes patients were identified. The objective of this study was to investigate differences in outpatient costs between pharmaceutically treated diabetes patients with and without depression.

### Ethics statement

The study has been conducted according to the principles expressed in the Declaration of Helsinki. The study was approved by the scientific council of the Primary Health Care Organisation Almere. The data were analyzed anonymously. Therefore, no ethical approval was necessary for this project.

### Data collection

Data were retrieved from electronic medical records kept by 20 general practices organized in one large primary care organization in Almere. Almere is a municipality in the centre region of the Netherlands with approximately 184,400 citizens (7 July 2008). All primary care practices and pharmacies in Almere are organized in one central health organization and share the same electronic medical record system saved in one central database. Data in this system include information on consultations, diagnoses, referrals and prescriptions. Diagnoses are recorded using the ICPC-2 coding system (International Classification of Primary Care). Drugs are classified according to the Anatomical Therapeutic Chemical (ATC) classification system. In the ATC classification system which is maintained by the WHO, the active substances of drugs are divided into different groups according to the organ or system on which they act and their chemical, pharmacological and therapeutic properties.

The Dutch health care system is based on the principle that everybody has adequate access to health care. Almost everyone in The Netherlands is registered with a general practitioner. Until 2006 two forms of insurance existed in The Netherlands: private and social insurance. People with earnings above approximately €30,000 per year and their dependants were excluded from statutory coverage provided by public sickness funds and could purchase coverage from private health insurers.

### Patient selection

**Figure 1:**
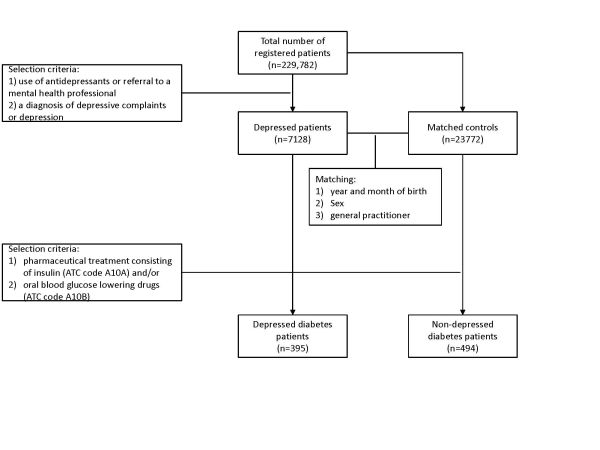


The patient selection procedure is depicted in Figure 1 and described here. In the study period (2002 to 2004), a total of 229,782 patients were registered at the 20 primary care practices. Depressed patients were selected according to the following criteria: 1) use of antidepressants in the period 2002-2004 (ATC code N06A) or referral to a mental health professional (psychiatrist, psychologist, social worker, psychotherapist or regional institute for mental welfare) and 2) a diagnosis of depressive complaints or depression (ICPC code P03 or P76). In this way, 7,128 treated depressed patients were identified. Controls consisting of registered patients at the same primary care practices as depressed patients. To ensure comparability between depressed patients and controls, controls were matched with depressed patients based on year and month of birth, sex, and general practitioner (GP). This resulted in 23,772 matched controls and a total study population of 30,900 patients. The number of matched controls per depressed patient varied with age from 4 in the age group 31-40 years to 1 in the age group > 90 years.

Subsequently, in this population of 30,900 primary care patients diabetes patients were identified. Patients were classified as having diabetes when they received pharmaceutical treatment consisting of insulin (ATC code A10A) and/or oral blood glucose lowering drugs (ATC code A10B). In this study sample, a diagnosis of diabetes was based on medication data and not on diagnostic codes. Medication data rather than problem lists were used, because problem lists (a list of clinically relevant, current health problems of the patient coded using the ICPC-2) may not be reliable and/or up to date. This means that it was not possible to discriminate between diabetes mellitus types 1 and 2 and that patients treated by lifestyle advices only are not included. A total of 393 (5.5%) depressed patients with diabetes was identified and 494 (2.1%) non-depressed patients with diabetes resulting in 1 to 2 non-depressed patients per depressed patient.

### Co-morbidity

To define co morbidity a Dutch adaptation [12] of the revised chronic disease score (CDS) was used [13]. The CDS is a measure of chronic disease status and uses ATC codes from automated pharmacy data as an indicator for the presence of certain chronic conditions. The following 25 conditions are distinguished: coronary and peripheral vascular disease, epilepsy, hypertension, HIV/AIDS, tuberculosis, rheumatologic conditions, hyperlipidemia, malignancies, Parkinson's disease, renal disease (including end stage renal disease), cardiac disease/arteriosclerotic cardiovascular disease/congestive heart failure, diabetes, glaucoma, peptic acid disease, cystic fybrosis, transplantations, respiratory illness/asthma, thyroid disorders, gout, Crohn's and ulcerative colitis, pain and inflammation, pain, depression, psychotic illness (including bipolar disorders), anxiety and tension. Diabetes and depression were excluded from the analyses concerning comorbidity.

### Health care utilization

The data that were extracted from the medical record system concerned only outpatient costs, which included GP care, referrals, and medication. Costs were not restricted to diabetes only but considered all causes. In case of a referral by the GP, data on the duration of treatment were lacking. Therefore, the average number of treatment sessions was obtained from reports on health care utilization of Dutch national organizations (Table 1) [14-22].

**Table 1:** Mean number of contacts and unit costs in Euros for the year 2003.

	Number of contacts	Unit cost (€, 2003)
Contacts with the GP		
Consultation*	--	20.20^a^
Consultation, long duration*	--	40.40^a^
Home visit*	--	40.40^a^
Home visit, long duration*	--	80.80^a^
Telephone contact*	--	10.10^a^
Repeat prescription*	--	10.10^a^
Physiotherapy^†‡^	11.0 or 13.1	22.75^a^
Dietician	5.0	14.07^b^
Mental health care		
Social worker^†^	7.5	47.86^c^
Psychologist^†^	13.0	76.00^a^
Psychiatrist^†^	13.0^§^	76.00^a^
Psychotherapist^†^	13.0^§^	76.00^a^
Regional institute for mental	16.0	124.00^a^
welfare^†^		
Outpatient clinic^||^	4.1	56.00^a^
Medication	--	dependent on drug & dose

* exact number of contacts available from the medical record system

^†^estimated number of sessions per referral

^‡^depending on type of insurance (private or public insurance)

^§ ^assumed to be the same as for a psychologist

^||^estimated number of sessions per patient per year

^a ^Dutch standard cost [21,22]

^b ^mean price obtained from professionals

^c ^price according to professional organisation

### Costs

Costs were calculated using Dutch guidelines for cost studies. If available, Dutch standard costs were used to value health care utilization [21,22]. Otherwise, prices from health care providers themselves, prices from professional organizations or tariffs were used. Medication costs were estimated using prices from the pharmacy database of the Royal Dutch Society for Pharmacy [23]. For drugs of which 300 units or more were delivered the specific price was obtained from the pharmacy database. For all other drugs the median of the obtained drug prices, which was to €0.19 per unit, was used. All prices were adjusted to the year 2003 using consumer price indices [20]. Table 1 lists the unit costs used in this cost-of-illness study.

### Analysis

In all analyses depressed patients with diabetes were compared with non-depressed patients with diabetes. Healthcare utilization was dichotomized according to whether a patient visited a specific provider yes or no. These rates were compared using logistic binary regression while correcting for age and sex. Costs generally have a highly skewed distribution caused by many patients with low costs and few patients with high costs [24]. Therefore, bias-corrected and accelerated bootstrapping with 1000 replications was used to compare costs between the two groups [25]. Bootstrapping was performed using StataSE 10 for Windows (Stata Corp LP, USA).

Patients in this study were clustered within GPs. It is reasonable to assume that patients between GPs differ in some characteristics, for example personality characteristics of the GP. Multilevel modelling is a statistical technique that can be used to correct for this clustering. The clustering in this study was modest (ICC 0.06), but we still decided to correct for clustering. Therefore, a random intercept was added to the multilevel models with group (depressed or non-depressed) as the central determinant to correct for clustering of patients within GPs. Total costs were adjusted for potential confounding by age, sex, type of insurance and medical comorbidity in the multilevel analyses to be able to estimate the effect of being depressed on outpatient costs among diabetes patients as unbiased as possible. All conditions distinguished by the CDS except depression and diabetes were included in the model as being present or not. In 2002-2004 patients could have private or social insurance. Private insurance indicates a higher socioeconomic status. Therefore, type of insurance was also included. The multilevel models were subsequently bootstrapped (1000 replications) and bias-corrected and accelerated confidence intervals were estimated. The multilevel analyses and bootstrapping were performed in StataSE 10 for Windows (Stata Corp LP, USA).

## Results

### Study sample

Depressed patients with diabetes were older and had more comorbid conditions than non-depressed patients with diabetes, whereas diabetes treatment (oral antidiabetics and insulin) did not differ (Table 2).

**Table 2:** Study sample characteristics, diabetes regimen and co morbidity of depressed patients with diabetes versus non-depressed patients with diabetes.

Characteristic		Depressed (n = 393)	Non-depressed (n = 494)
Mean age	Years (SD)	59.1 (14.6)	53.1 (13.5)
Sex	% Female (*n*)	61.1 (240)	59.9 (296)
Private insurance	% (*n*)	10.7 (42)	15.4 (76)
Mental health treatment			
No mental health treatment	% (*n*)	0 (0)	86.4 (427)
Antidepressants	% (*n*)	62.1 (244)	7.3 (36)
Referral to a psychiatric care provider	% (*n*)	11.5 (45)	4.5 (22)
Antidepressants + referral	% (*n*)	26.5 (104)	1.8 (9)
Diabetes regimen			
Oral glucose medication	% (*n*)	51.1 (201)	50.6 (250)
Insulin	% (*n*)	27.2 (107)	30.2 (149)
Oral glucose medication + insulin	% (*n*)	21.6 (85)	19.2 (95)
Comorbidity			
0 comorbid conditions	% (*n*)	1.8 (7)	9.9 (49)
1-2 comorbid conditions	% (*n*)	19.6 (77)	38.7 (191)
More than 3 comorbid conditions	% (*n*)	78.6 (309)	51.4 (254)

### Health care utilization

Table 3 shows the health care utilization rates in depressed and non-depressed patients with diabetes. Depressed patients with diabetes were more often referred to all health care providers than non-depressed patients with diabetes. Ninety-eight percent of all patients in both groups visited their GP at least once in 2002-2004. Significantly more depressed patients with diabetes were referred to an outpatient clinic during the three years of the study than non-depressed patients with diabetes (89% versus 78%, respectively; OR = 2.5, *p *< 0.001). Thirty percent of depressed patients with diabetes were referred to a mental health care provider or institution compared with 5% of non-depressed patients with diabetes in 2002-2004 (OR = 10.7, *p *< 0.001).

**Table 3:** Mean (SD) health care utilization per patient during 3 years (2002-2004) in depressed patients with diabetes and non-depressed patients with diabetes.

Category	Depressed (n = 393)	Non-depressed (n = 494)
Contacts with the GP		
Consultation	18.9 (14.3)	13.5 (10.6)
Home visit	3.0 (7.7)	0.7 (2.6)
Telephone contact	5.1 (9.8)	2.1 (3.3)
Repeat prescription	31.5 (26.6)	20.2 (19.3)
Physiotherapy	13.3 (20.7)	8.7 (16.3)
Dietician	0.1 (1.3)	0.1 (1.5)
Mental health care		
Social worker	0.7 (2.5)	0.2 (1.4)
Psychologist	0.1 (1.3)	0.0 (0.6)
Psychiatrist	7.0 (15.9)	0.8 (5.8)
Psychotherapist	0.1 (0.9)	0 (0)
Regional institute for mental	0.2 (2.1)	0.1 (1.4)
welfare		
Outpatient clinic	8.0 (4.1)	6.4 (4.4)
Medication deliveries	158.2 (191.6)	87.4 (131.0)

### Costs

Mean costs per year in the period 2002-2004 and standard deviations are presented in Table 4. Costs in all categories were significantly higher in depressed patients with diabetes than in non-depressed patients with diabetes. Mean total outpatient costs per year in depressed patients with diabetes were €1039 (743) and more than two times as high as in non-depressed patients with diabetes (€492, SD 434). Mean total outpatient costs increased with an increasing number of comorbid conditions. Costs in depressed patients with diabetes were statistically significantly higher than in non-depressed patients with diabetes (mean difference €547, 95% CI 466 to 635). Mental health care costs accounted for one third of this difference in costs (mean difference €174, 95% CI 134 to 224).

**Table 4:** Mean (SD) annual costs (unadjusted) in depressed patients with diabetes and non- depressed patients with diabetes.

Cost category	Depressed	Non-depressed	Difference Unadjusted (95% CI)	Difference adjusted (95% CI)*
GP costs	304 (218)	182 (133)	122 (97; 145)	103 (81; 129)
Physical therapy	102 (158)	68 (125)	34 (17; 55)	33 (14; 53)
Dietician	101 (157)	66 (124)	35 (16; 55)	34 (16; 55)
Mental health care	202 (440)	28 (179)	174 (134; 224)	194 (151; 247)
Outpatient clinic	149 (76)	119 (82)	30 (19; 41)	31 (21; 42)
Medication	182 (247)	30 (160)	152 (125; 182)	157 (129; 186)
Total costs	1039 (743)	492 (434)	547 (466; 635)	553 (468; 641)

* Differences were adjusted for age and sex.

Table 5 shows mean total outpatient costs per year in the period 2002-2004 in non-depressed patients with diabetes (constant) and the difference in mean total costs between depressed and non-depressed patients with diabetes (β_depression_) after correction for possible confounders in the multilevel analyses. Correction for comorbidity had the largest impact on the difference in costs between depressed and non-depressed patients with diabetes. When comorbidity was divided into three distinct categories the difference in total annual costs between depressed and non-depressed patients with diabetes changed from €539 (uncorrected, 95% CI 469 to 634) to €463 (corrected, 95% CI 386 to 554).

**Table 5:** Results of multilevel analyses incorporating a random intercept for clustering of patients within general practitioners.

Model	Constant	β_depression_* (SE)	95% CI
*Crude model*	493	539 (41)	469; 634
Only group included			
*Model 1*	521	548 (42)	473; 642
Crude model + age, sex			
*Model 2*	544	542 (43)	461; 634
Model 1 + type of insurance			
*Model 3*	490	540 (43)	459; 634
Model 2 + diabetes treatment			
*Model 4*	370	408 (42)	331; 494
Model 3 + comorbidity^1^			
*Model 5*	365	463 (42)	386; 554
Model 3 + comorbidity in three categories^2^			

* Difference in total annual costs between depressed patients with diabetes and non-depressed patients with diabetes

^1 ^Limited set of selected comorbidities yes or no: coronary and peripheral vascular disease, hypertension, rheumatologic conditions, hyperlipidemia, cardiac disease, peptic acid disease, respiratory illness, pain and inflammation, pain, and anxiety and tension.

^2 ^No comorbidity, 1 or 2 comorbid conditions, and 3 or more comorbid conditions

## Discussion

In this study we identified diabetes patients in a primary care cohort consisting of treated depressed patients and non-depressed matched controls. We found that total costs in depressed patients with diabetes were twice as high as in non-depressed patients with diabetes in the Netherlands. Even after correction for age, sex, type of insurance, diabetes treatment, and comorbid conditions total annual costs in depressed patients were €463 higher than in non-depressed patients, which was statistically significant. Adjustment for comorbidity had the largest impact on the difference in costs between the two groups.

Consistent with previous research, our results show that depression comorbid with diabetes was associated with significantly higher health care costs than diabetes alone and that this increase was mainly the result of increased general medical care and not mental health care utilization [9,10]. Our study is different from these studies in the sense that we identified diabetes patients in a large cohort of depressed primary care patients and their matched controls, while these other studies assessed the presence of depression using questionnaires in participants sampled from a diabetes registry. The observed increase in health care costs in depressed patients with diabetes may be caused by unobserved differences in severity of diabetes or other comorbid chronic conditions [9]. However, even after we corrected for comorbid conditions health care costs in depressed patients with diabetes were significantly higher than in non-depressed patients with diabetes. An alternative hypothesis is that depression may interfere with effective self-care for diabetes leading to higher health care costs [26] and that effective treatment may result in normalised health care costs. Recent studies evaluating the cost-effectiveness of systematic identification and treatment of depression in diabetes patients showed that such interventions may be cost-effective [27,28]. The Pathways Study showed that a collaborative depression care program resulted in lower costs over a 5-year period compared with usual care, although this difference was not statistically significant [29].

The cost estimates obtained in our study are lower than in other studies [30,31]. The most likely explanation for this difference is that, since these other studies depended on GPs for patient selection, patients with more severe diabetes were included in these studies. Other explanations may be that not all referrals were registered by the GPs in their electronic medical record system and that the national health care utilization rates may be an underestimation of the number of contacts for diabetes patients.

Strengths of this study are that we used complete records of a large cohort of depressed patients and matched controls in which we identified diabetes patients. Second, ATC codes of delivered medication were used to determine the most important covariates, i.e., diabetes, diabetes treatment and comorbidities. We expect that this information on delivered medication is very accurate, since reimbursement by insurance companies is based on the same data we used for this study. However, when interpreting these findings one should also consider some limitations. Only outpatient costs were considered in this study. However, hospitalization costs and lost productivity costs due to absenteeism, presenteeism, early retirement and mortality may be substantially larger than outpatient costs in this population. From a societal perspective, this is an important limitation. Including these costs will result in higher cost estimates, and, most likely, to a larger difference in costs between depressed and non-depressed diabetes patients. Other limitations include the following. First, it was not possible to discriminate between diabetes mellitus type 1 and type 2, because we used medication data to determine the presence of diabetes. This means that this study sample does not include diabetes patients treated by lifestyle advices only, which is likely to underestimate the true diabetes prevalence rates in both groups. Second, we only had the disposal of data on referrals and not on the number of contacts following a referral. Therefore, we had to use national health care utilization data to estimate the number of contacts patients had with other health care providers than the GP. Patients with depression and diabetes are likely to require more complex care than patients with diabetes alone. However, the magnitude of this effect is unknown. Therefore, the cost difference reported in this study is likely to be an underestimation of the true cost difference. Third, comorbidity was defined based on medication prescriptions. Thus, it is possible that patients who incidentally used one of the medications included in the CDS are incorrectly labelled as having a chronic disease. This may have overestimated the effect of comorbidity on total costs. Fourth, for our selection of depressed patients, we relied on the diagnostic accuracy and treatment by the GP. Thus, our study did not include depressed persons who do not visit their GP, depressed persons who are not recognized as being depressed by the GP and depressed persons who are recognized as being depressed by the GP but do not receive a registered diagnosis of depression or treatment for their depression. Therefore, total costs of depression among patients with diabetes are underestimated, while inclusion of depressed persons in matched controls may lead to an overestimation of mean costs in controls. Finally, diabetes patients were identified in depressed patients and non-depressed matched controls. More than one control was available for depressed patients in younger age categories. As the prevalence of diabetes increases with age and depressed patients were older than non-depressed patients as a consequence of our selection process, it is possible that the prevalence of diabetes in depressed patients is an overestimation.

In conclusion, our results show that even after adjustment for comorbid conditions health care costs in depressed patients with diabetes are significantly higher than in non-depressed patients with diabetes. More longitudinal research in usual clinical practice is needed on the costs of depression in combination with diabetes with a distinction into diabetes mellitus 1 and 2. Research should also indicate whether adequate treatment of depression among diabetes patients can lead to lower health care costs in the long term. Finally, the results are of importance to policy makers. The results of the study clearly show that the combination of diabetes and depression leads to markedly increased health care costs. Considering the expected rise in the prevalence of diabetes, funding should be made available to disentangle the relationship between diabetes and depression and to develop treatments to prevent depression in diabetes patients.

## Competing interests

The authors declare that they have no competing interests.

## Authors' contributions

J.E.B. researched the data. Both J.E.B and M.C.A interpreted the data, were involved in drafting the manuscript, revising it critically for important intellectual content and have given final approval of the version to be published. Both authors read and approved the final manuscript.

## Pre-publication history

The pre-publication history for this paper can be accessed here:

http://www.biomedcentral.com/1472-6963/12/46/prepub
